# Fenofibrate as a Modulator of the Renin–Angiotensin System in Su/Hx-Induced Pulmonary Arterial Hypertension

**DOI:** 10.3390/ijms262110251

**Published:** 2025-10-22

**Authors:** Karla M. Rada-Pascual, Alejandra M. Zúniga-Muñoz, Yamnia Q. Alvarez-Alvarez, Leonardo Del Valle-Mondragón, Ivan Rubio-Gayosso, Constanza E. Martínez-Olivares, Rogelio Hernández-Pando, Horacio Osorio-Alonso, José L. Sánchez-Gloria, Pedro L. Flores, Julio Sandoval, Jaime H. Gómez-Zamudio, Roxana Carbó, Fausto Sánchez-Muñoz

**Affiliations:** 1 Departamento de Fisiología, Instituto Nacional de Cardiología Ignacio Chávez, Mexico City 14080, Mexico; michelrp36@gmail.com (K.M.R.-P.); yamniaalvarezalvarez@gmail.com (Y.Q.A.-A.); 2 Sección de Estudios de Posgrado, Escuela Superior de Medicina, Instituto Politécnico Nacional, Mexico City 11340, Mexico; aiorubio@gmail.com; 3 Departamento de Biomedicina Cardiovascular, Instituto Nacional de Cardiología Ignacio Chávez, Mexico City 14080, Mexico; mvzalemar@yahoo.com.mx; 4 Departamento de Farmacología “Dr. Rafael Méndez Martínez”, Instituto Nacional de Cardiología Ignacio Chávez, Mexico City 14080, Mexico; leonardo.delvalle@cardiologia.org.mx; 5 Sección de Patología Experimental, Departamento de Patología, Instituto Nacional de Ciencias Médicas y Nutrición “Salvador Zubirán”, Mexico City 14080, Mexico; constanzamtz@exalumno.unam.mx (C.E.M.-O.); rogelio.hernandezp@incmnsz.mx (R.H.-P.); 6 Departamento de Fisiopatología Cardio-Renal, Instituto Nacional de Cardiología Ignacio Chávez, Mexico City 14080, Mexico; horacio.osorio@cardiologia.org.mx; 7 Division of Nephrology, Department of Internal Medicine, Rush University Medical Center, Chicago, IL 60612, USA; jose_sanchez@rush.edu; 8 Departamento de Instrumentación Electromecánica, Instituto Nacional de Cardiología Ignacio Chávez, Mexico City 14080, Mexico; lorenzopelofec@yahoo.com.mx; 9 Departamento de Inmunología, Instituto Nacional de Cardiología Ignacio Chávez, Mexico City 14080, Mexico; sandovalzarate@prodigy.net.mx; 10 Unidad de Investigación Médica en Bioquímica, Hospital de Especialidades “Bernardo Sepulveda”, Centro Médico Nacional Siglo XXI, Instituto Mexicano del Seguro Social, Mexico City 06720, Mexico; jaime.gomezz@imss.gob.mx

**Keywords:** pulmonary arterial hypertension, fenofibrate, renin–angiotensin system

## Abstract

We evaluated the effects of fenofibrate (FF) in a SU5416/hypoxia model of pulmonary arterial hypertension (PAH) with a specific focus on its influence on the renin–angiotensin system (RAS). We assessed right ventricular systolic pressure (RVSP), mean pulmonary artery pressure (mPAP), medial pulmonary artery wall thickening, right ventricular (RV) hypertrophy, systolic pulmonary artery pressure (SPAP), pulmonary artery effective elastance (PAEa), RV diastolic pressure (RVDP), RV developed pressure (RVDevP), right ventricular–pulmonary arterial coupling index (RVPAC), RV dp/dt max and dp/dt min. Levels of angiotensin II, angiotensin (1–7), angiotensin-converting enzyme 2 (ACE2), *Bmpr2*, *Smad5* and nitrite (NO_2_^−^) and nitrate (NO_3_^−^) in the lung and RV were evaluated. The expression of AT1R, MAS receptors, and ACE2 in lung tissue was assessed. FF prevented the increase in RVSP, mPAP, RV hypertrophy, reduced pulmonary arterioles remodeling, and attenuated the rise in SPAP, mPAP, and PAEa. In the RV, it reduced RVDevP and prevented the decrease in dp/dt min, without affecting RVDP. RVPAC showed partial improvement. In lung tissue, FF decreased angiotensin II levels, the Ang II/Ang-(1–7) ratio, and reduced angiotensin II receptor type 1 (AT1R) expression, while preserving the receptor for the angiotensin-(1–7) (MAS) and ACE2. FF tended to restore *Bmpr2*/*Smad5* expression. NO_2_^−^ levels were preserved and tended to preserve (NO_3_^−^) levels. In the RV, Ang-(1–7) increased, ACE2 was preserved, and NO_2_^−^ and NO_3_ levels were maintained. FF exerts protective effects in Su/Hx-induced PAH.

## 1. Introduction

Pulmonary arterial hypertension (PAH) is an infrequent, gradual, multifactorial, and potentially fatal cardiopulmonary disease [1,2,3,4]. PAH clinical manifestations originate from a gradual increase in pulmonary vascular resistance (PVR), primarily due to remodeling of the pulmonary vasculature [5]. These structural alterations in the pulmonary arteries lead to a continuous increase in pulmonary arterial pressure (PAP) and PVR [6]. The persistent pressure overload imposes an increased stress on the right ventricle wall, resulting in hypertrophy and ultimately failure of the right ventricle, which is the leading cause of death [7,8]. PAH pathogenesis involves multiple pathways, including mutations in the bone morphogenetic protein receptor type II (*BMPR2*), nitric oxide (NO) metabolism, and dysregulation of the renin–angiotensin system (RAS) [1,9,10,11,12,13]. Both experimental models and patient studies have demonstrated a decrease in BMPR2 and SMAD5 expression. Likewise, reduced nitrate (NO_3_^−^) and nitrite (NO_2_^−^) levels have been consistently observed, particularly in individuals with identified BMPR2 gene alterations [14,15,16]. Dysfunction in BMPR2 signaling and reduced NO bioavailability generate an altered endothelial phenotype that favors smooth muscle cell proliferation, endothelial apoptosis, inflammation, and increased vascular tone [11,12,13,17]. These processes converge in the progression of PAH and have been associated with increased clinical severity and worse prognosis [5,18]. Clinical and preclinical studies have described both systemic and local activation of the RAS, including the pulmonary vasculature and right ventricle, in PAH [9,10,19,20,21,22,23,24,25,26,27,28]. In the lung tissue of PAH patients, overexpression of angiotensin II (Ang II), angiotensin-converting enzyme (ACE), and the angiotensin II type 1 receptor (AT1R) has been observed. In contrast, a reduction in angiotensin-converting enzyme type 2 (ACE2) and angiotensin (1–7) (Ang-(1–7)) has been reported [9,10,22,25]. This imbalance promotes the activity of the vasoconstrictor ACE/Ang II/AT1R axis over the vasoprotective ACE2/Ang-(1–7)/MAS axis. Furthermore, localized increases in ACE expression have been reported in the right ventricle in rats under chronic hypoxia conditions [23]. RAS imbalance in PAH has been primarily associated with pulmonary arterial smooth muscle cell (PASMC) proliferation and sustained vasoconstriction, resulting in increased mean pulmonary arterial pressure and pulmonary vascular resistance. These processes accelerate progression to right ventricular failure and reflect greater disease severity [19,27,29].

Current therapeutic strategies for PAH focus on improving pulmonary hemodynamics and prolonging progression-free survival [30]. However, despite pharmacological advances, substantial improvements in long-term survival have not been achieved. Therefore, exploring novel pharmacological alternatives for treating this disease is crucial.

Fenofibrate (FF) is a commonly used dyslipidemia drug that exerts its primary effects on genes involved in lipid metabolism [31,32,33]. Several studies have shown that FF exerts antihypertrophic and antifibrotic effects, promotes NO production, and improves endothelial function, suggesting that it may have potential applications in the management of cardiovascular and pulmonary pathologies [33,34,35,36]. In recent years, FF has been investigated for its potential role in modulating the RAS pathway. In experimental models of metabolic syndrome and myocardial ischemia, FF treatment has been shown to regulate both the classical (ACE/Ang II/AT1R) and non-classical (ACE2/Ang-(1–7)/AT2R/MAS) RAS axes, primarily in cardiac tissue [34]. This was evidenced by a decrease in Ang II levels and a reduction in the expression of its receptor, AT1R. Concurrently, FF increased the expression of ACE2 and the levels of Ang-(1–7), thus promoting the activation of the ACE2/Ang-(1–7)/AT2R/MAS axis, which is known for its hemodynamic-modulating, antifibrotic, and anti-inflammatory effects [37,38,39]. Additionally, FF treatment inhibits the activation of the Ang III/Ang IV/insulin-regulated aminopeptidase (IRAP) axis, an emerging branch of the RAS implicated in inflammatory processes [37]. Also, Vera et al. demonstrated that FF lowers mean arterial pressure in a model of Ang II-induced systemic hypertension, providing evidence supporting its potential as a RAS modulator in cardiovascular conditions [40]. In the context of PAH, Galhotra et al. demonstrated that FF administration prevents monocrotaline (MCT)-induced PAH by the reduction in oxidative stress and inflammation, as well as the inhibition of NADPH oxidase (NOX) expression in lung tissue [7]. The available evidence suggests that FF may exert beneficial effects on Su/Hx-induced PAH by modulating the RAS. Therefore, the present study aimed to evaluate the impact of FF on Su/Hx-induced PAH, with a specific focus on its influence on RAS.

## 2. Results

### 2.1. Validation of the Su/Hx-Induced PAH Model and the Protective Effects of Fenofibrate

To evaluate the establishment of PAH in the Su/Hx model and the protective effect of FF administration, we analyzed hemodynamic and structural parameters characteristic of the disease. No statistically significant differences in final body weight or absolute weight gain were observed in the experimental groups (Supplementary Figure S1).

**Figure 1:**
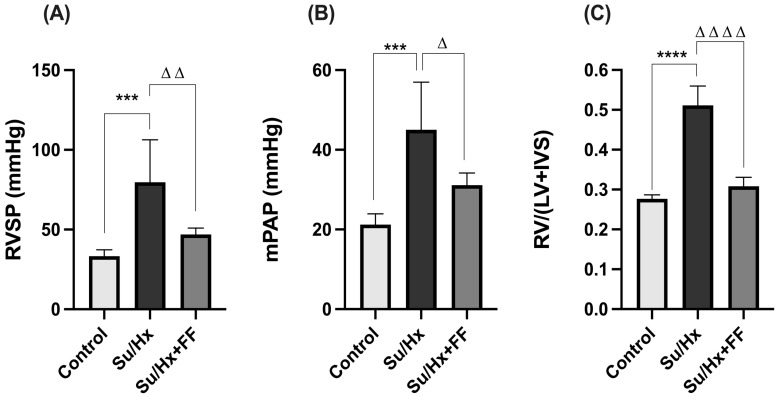
Fenofibrate attenuates the development of Su/Hx-induced PAH. (**A**) Right ventricular systolic pressure (RVSP), (**B**) mean pulmonary artery pressure (mPAP), (**C**) right ventricular hypertrophy. Data are presented as mean ± SD. Differences were tested by one-way ANOVA followed by Tukey’s multiple comparisons post hoc test; *p* ≤ 0.05 was considered statistically significant. *** *p* < 0.001, **** *p* < 0.0001 vs. Control; Δ *p* < 0.05, ΔΔ *p* < 0.01, ΔΔΔΔ *p* < 0.0001 vs. Su/Hx.

First, rats exposed to Su/Hx showed a significant increase in the RVSP (*p* = 0.0004 vs. control). This increase was prevented by FF administration (*p* = 0.0070 vs. Su/Hx; Figure 1A). Similarly, mPAP was significantly increased in the Su/Hx group compared to control (*p* = 0.0007); this effect was attenuated after FF administration (*p* = 0.0275 vs. Su/Hx; Figure 1B). Consistent with these findings, right ventricular hypertrophy was also assessed. Rats in the Su/Hx group developed right ventricular hypertrophy compared to the control group (*p* < 0.0001), whereas FF administration prevented the development of right ventricular hypertrophy (Figure 1C).

### 2.2. Fenofibrate Administration Attenuates Medial Wall Thickening in Pulmonary Arterioles in a Su/Hx-Induced PAH Model

**Figure 2:**
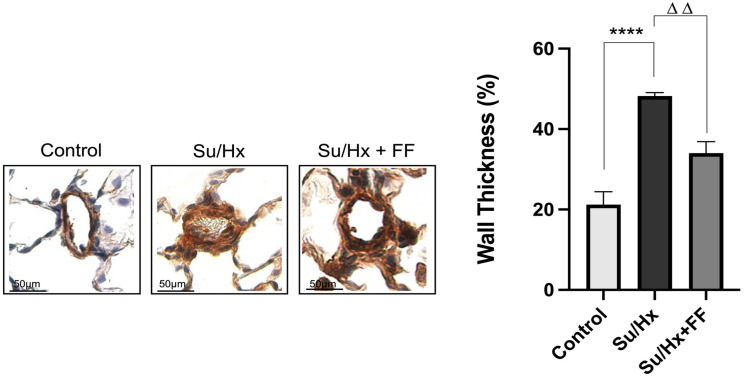
Fenofibrate attenuates thickening of the medial wall of pulmonary arterioles. Representative micrographs of immunohistochemical labeling of actin myofilaments of smooth muscle cells that constitute the medial arterioles’ wall. Compared to the control group, SuHx-treated rats exhibit medial layer thickening and a narrowed lumen; the medial layer is thinner in SuHx rats with FF administration. This difference was confirmed by morphometry, which showed significantly less widening of the arterioles’ muscular wall in the Su/Hx + FF rats than in Su/Hx rats. Data are presented as mean ± SD. Differences were tested by one-way ANOVA followed by Tukey’s multiple comparisons post hoc test; *p* ≤ 0.05 was considered statistically significant. *n* = 3. **** *p* < 0.0001 vs. Control; ΔΔ *p* < 0.01 vs. Su/Hx.

Histological analysis showed a significant thickening of the media muscular wall of pulmonary arterioles associated with a narrowing of the vascular lumen in Su/Hx rats relative to controls (*p* < 0.0001). FF administration significantly attenuated this effect by approximately 50% compared to the Su/Hx group (*p* = 0.0011 vs. Su/Hx; Figure 2).

### 2.3. Fenofibrate Administration Improves Pulmonary Hemodynamics in the Su/Hx-Induced PAH Model

**Figure 3:**
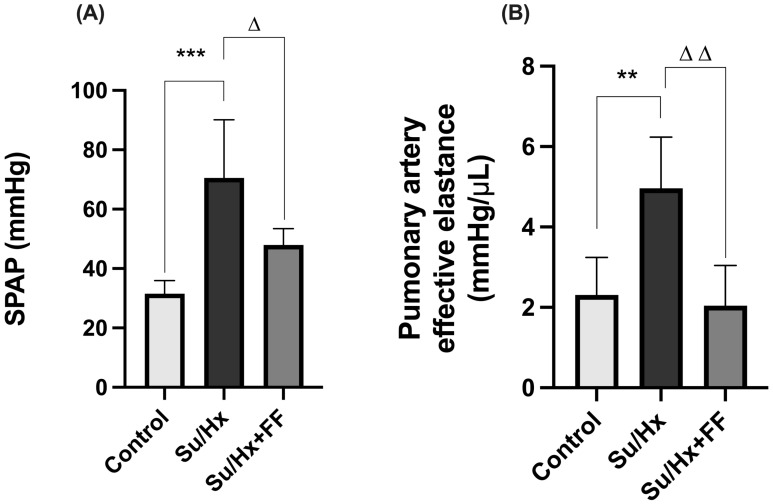
Fenofibrate improves pulmonary hemodynamic parameters in PAH. (**A**) Systolic pulmonary artery pressure (SPAP), (**B**) pulmonary artery effective elastance. Data are presented as mean ± SD. Differences were tested by one-way ANOVA followed by Tukey’s multiple comparisons post hoc test; *p* ≤ 0.05 was considered statistically significant. ** *p* < 0.01, *** *p* < 0.001 vs. Control; Δ *p* < 0.05, ΔΔ *p* < 0.01 vs. Su/Hx.

We evaluated the effect of FF on pulmonary hemodynamics, as shown in Figure 3. Rats exposed to Su/Hx showed a significant increase in systolic pulmonary artery pressure (SPAP) (*p* = 0.0007 vs. control); this increase was attenuated by FF administration (*p* = 0.0299 vs. Su/Hx; Figure 3A). Furthermore, PAEa was higher in the Su/Hx group (*p* = 0.0056 vs. control); this elevation was prevented by FF administration (*p* = 0.0028 vs. Su/Hx; Figure 3B).

We evaluated the involvement of RAS in the Su/Hx-induced PAH model and the effect of FF on this system. We assessed Ang II, Ang (1–7), and ACE2 concentrations, as well as the Ang II/Ang (1–7) ratio and the AT1R receptor, MAS receptor, and ACE2 expression in lung tissue.

### 2.4. Effect of Fenofibrate Administration on Renin–Angiotensin System Components in Lung Tissue in the Su/Hx-Induced PAH Model

**Figure 4:**
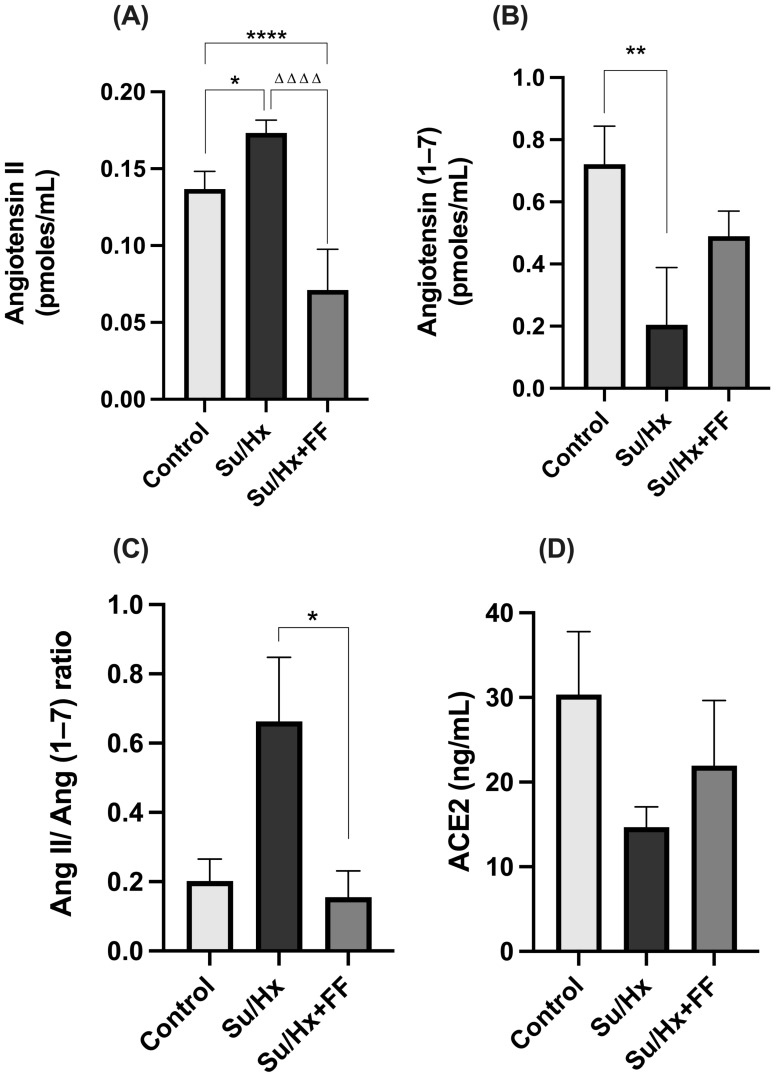
Fenofibrate modulates renin–angiotensin system components in lung tissue in PAH. (**A**) Angiotensin II (Ang II) concentrations. (**B**) Angiotensin (1–7) (Ang (1–7)) concentrations. (**C**) Ang II/Ang (1–7) ratio. (**D**) Angiotensin-converting enzyme 2 (ACE2) concentrations. Data are presented as mean ± SD. Differences were tested by one-way ANOVA followed by Tukey’s multiple comparisons post hoc test; *p* ≤ 0.05 was considered statistically significant. * *p* < 0.05, ** *p* < 0.01, **** *p* < 0.0001 vs. Control; ΔΔΔΔ *p* < 0.0001 vs. Su/Hx.

In lung tissue, we observed an increase in Ang II concentrations in the Su/Hx group compared to the control group (*p* = 0.0174). This increase was significantly reduced after FF administration (*p* < 0.0001; Figure 4A). As for Ang (1–7) concentrations, a significant decrease was observed in the Su/Hx group compared to the control group (*p* = 0.0051; Figure 4B). Unexpectedly, no significant changes were found in the Su/Hx + FF group. Nevertheless, the Ang II/Ang (1–7) ratio was significantly increased in the Su/Hx group compared to the control group (*p* = 0.0491); this increase was prevented by FF administration (*p* = 0.0073; Figure 4C). Additionally, ACE2 concentrations were evaluated, and no significant differences were found (Figure 4D).

### 2.5. Effect of Fenofibrate Administration on Pulmonary Expression of AT1R, MAS Receptor, and ACE2 in Lung Tissue in the Su/Hx-Induced PAH Model

**Figure 5:**
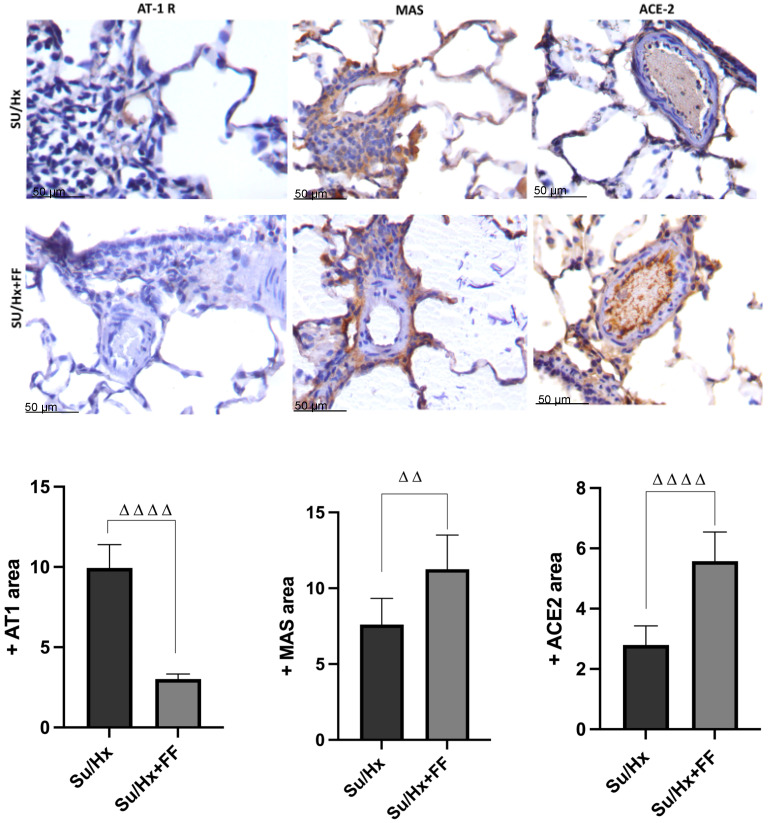
Fenofibrate effect on renin–angiotensin system components in lung tissue in PAH. Representative micrographs of renin–angiotensin system components detected by immunohistochemistry. They showed the positive area in pulmonary tissue comparing the Su/Hx and Su/Hx + FF experimental groups. The indicated molecules were highly expressed, particularly in the endothelial cells of hypertrophic arterioles in the Su/Hx + FF group, except for angiotensin II receptor type 1 (AT1R), which was more abundant in the Su/Hx group. (All micrographs 200×magnification). MAS: receptor for the angiotensin-(1–7); ACE2: Angiotensin-converting enzyme 2. ΔΔ *p* < 0.01, ΔΔΔΔ *p* < 0.0001 vs. Su/Hx.

Next, we evaluated other components of RAS, including the AT1R receptor, MAS receptor, and ACE2, in lung tissue by immunohistochemistry (Figure 5). The results showed an overexpression of the AT1R receptor in the endothelial cells of the arterioles in the Su/Hx group, which was reduced after FF administration. Regarding the MAS receptor, overexpression was observed in the smooth muscle cells and endothelial cells of the arterioles of the Su/Hx + FF group. At the same time, a lower expression was detected in the Su/Hx group. Finally, concerning ACE2, overexpression was observed in the endothelium and some alveolar epithelial cells and lymphocytes from the Su/Hx + FF group, and lower expression was detected in the Su/Hx group.

### 2.6. Effect of Fenofibrate Administration on Nitrate (NO_3_^−^) and Nitrite (NO_2_^−^) Concentrations in Lung Tissue in a Su/Hx-Induced PAH Model

**Figure 6:**
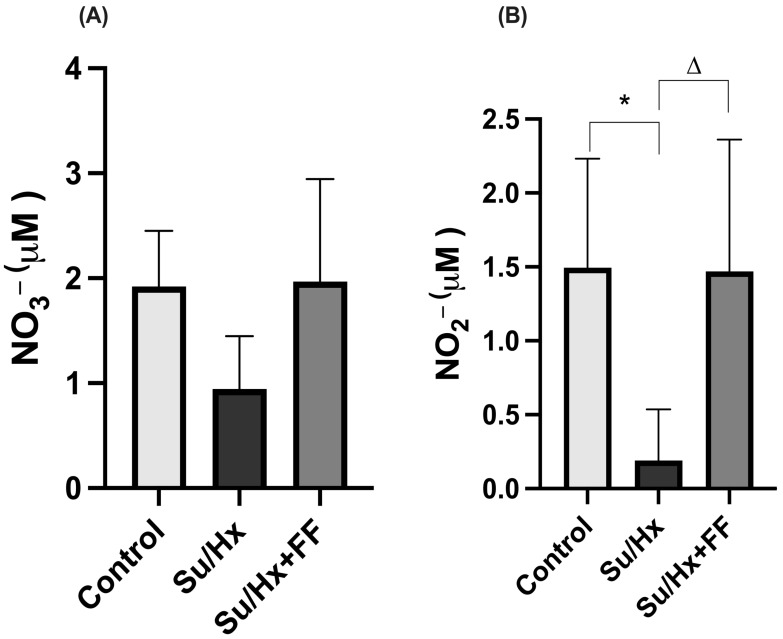
Fenofibrate preserves nitrate (NO_3_^−^) and nitrite (NO_2_^−^) concentrations in lung tissue. (**A**) NO_3_^−^ concentrations in lung tissue. (**B**) NO_2_^−^ concentrations in the lung tissue. Data are presented as mean ± SD. Differences were tested by one-way ANOVA followed by Tukey’s multiple comparisons post hoc test; *p* ≤ 0.05 was considered statistically significant. * *p* < 0.05 vs. Control; Δ *p* < 0.05 vs. Su/Hx.

In lung tissue, a decreasing trend in NO_3_^−^ concentrations was observed in the Su/Hx group compared to the control group; however, the difference did not reach statistical significance. Similarly, the Su/Hx + FF group partially recovered from NO_3_^−^ levels, although without achieving statistically significant differences (Figure 6A). As shown in Figure 6B, NO_2_^−^ concentrations were reduced in the Su/Hx group compared to the control group (*p* = 0.0298). FF administration preserved NO_2_^−^ concentrations compared to the Su/Hx group (*p* = 0.0331).

### 2.7. Effect of Fenofibrate Administration on Bmpr2 and Smad5 Expression in Lung Tissue in the Su/Hx-Induced PAH Model

**Figure 7:**
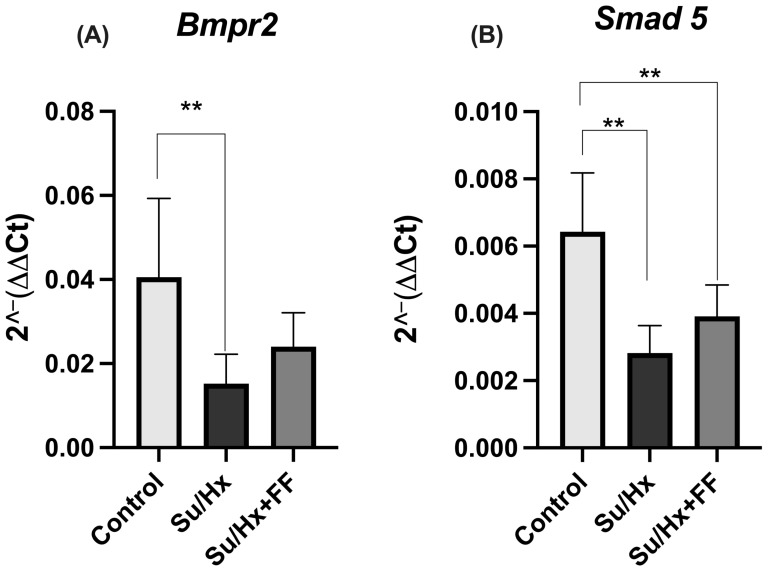
Fenofibrate partially preserves *Bmpr2* and *Smad5* expression in lung tissue in PAH. (**A**) Bone morphogenetic protein receptor type 2 (*Bmpr2*) gene expression levels. (**B**) Mothers against decapentaplegic homolog 5 (*Smad5*) gene expression levels. Data are presented as mean ± SD. Differences were tested by one-way ANOVA followed by Tukey’s multiple comparisons post hoc test; *p* ≤ 0.05 was considered statistically significant. ** *p* < 0.01 vs. Control.

As shown in Figure 7A, *Bmpr2* expression levels were significantly lower in the Su/Hx group compared with the control group (*p* = 0.0088). Although the Su/Hx + FF group showed a partial restoration, the difference was not statistically significant compared to the Su/Hx group. Concerning *Smad5* expression, a reduction was observed in the Su/Hx group compared to the control group (*p* = 0.0005). In the FF administration group, a trend towards increased *Smad5* levels was observed compared to the Su/Hx group; however, this recovery remained significantly below the levels observed in the control group (*p* = 0.0096; Figure 7B).

### 2.8. Effect of Fenofibrate Administration on Cardiac Hemodynamics in the Su/Hx-Induced PAH Model

**Figure 8:**
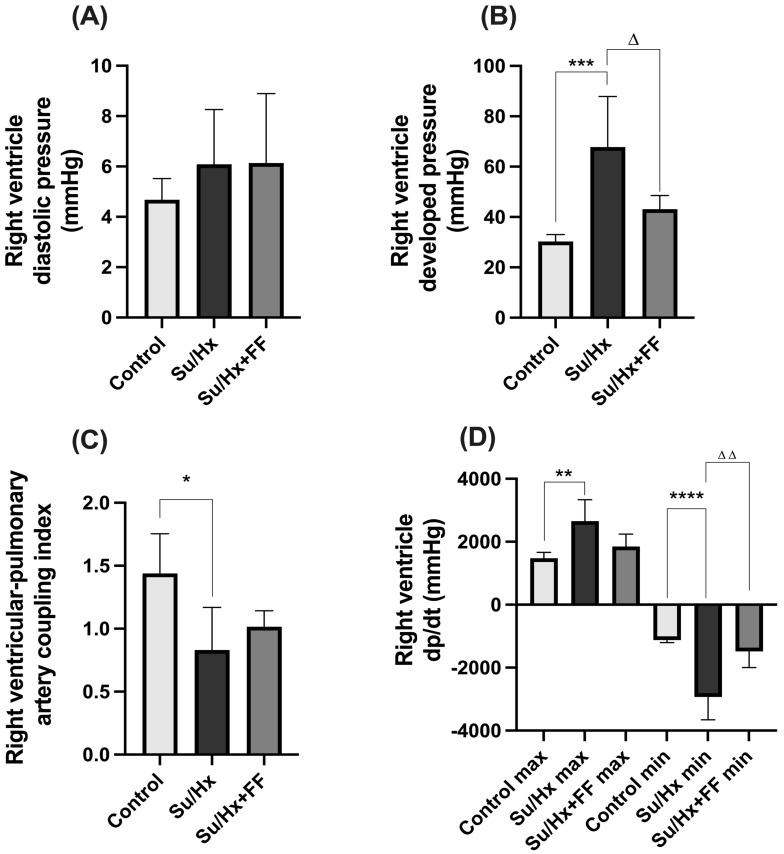
Fenofibrate improves right ventricular function in PAH. (**A**) Right ventricular diastolic pressure, (**B**) right ventricular developed pressure, (**C**) right ventricle–pulmonary artery coupling index, and (**D**) maximal and minimal rates of pressure change (dp/dt max and dp/dt min) in the right ventricle. Data are presented as mean ± SD. Differences were tested by one-way ANOVA followed by Tukey’s multiple comparisons post hoc test; *p* ≤ 0.05 was considered statistically significant. * *p* < 0.05, ** *p* < 0.01, *** *p* < 0.001, **** *p* < 0.0001 vs. Control; Δ *p* < 0.05, ΔΔ *p* < 0.01 vs. Su/Hx.

No significant changes were observed in RVDP, as shown in Figure 8A. In contrast, RVDevP was significantly increased in the Su/Hx group (*p* = 0.0010 vs. control); this increase was attenuated after FF administration (*p* = 0.0187 vs. Su/Hx; Figure 8B). Similarly, the RVPAC was significantly reduced in the Su/Hx group (*p* = 0.0165 vs. control). Although the Su/Hx + FF group showed partial restoration, the difference was not statistically significant compared to the Su/Hx group. Regarding contractility indices, exposure to Su/Hx induced a substantial increase in dp/dt max (*p* = 0.0098 vs. control), which was not modified by FF administration. However, the decrease in dp/dt min observed in the Su/Hx group (*p* < 0.0001 vs. control) was significantly prevented after FF administration (*p* = 0.0012 vs. Su/Hx; Figure 8D).

### 2.9. Effect of Fenofibrate Administration on Renin–Angiotensin System Components in the Right Ventricle in the Su/Hx-Induced PAH Model

**Figure 9:**
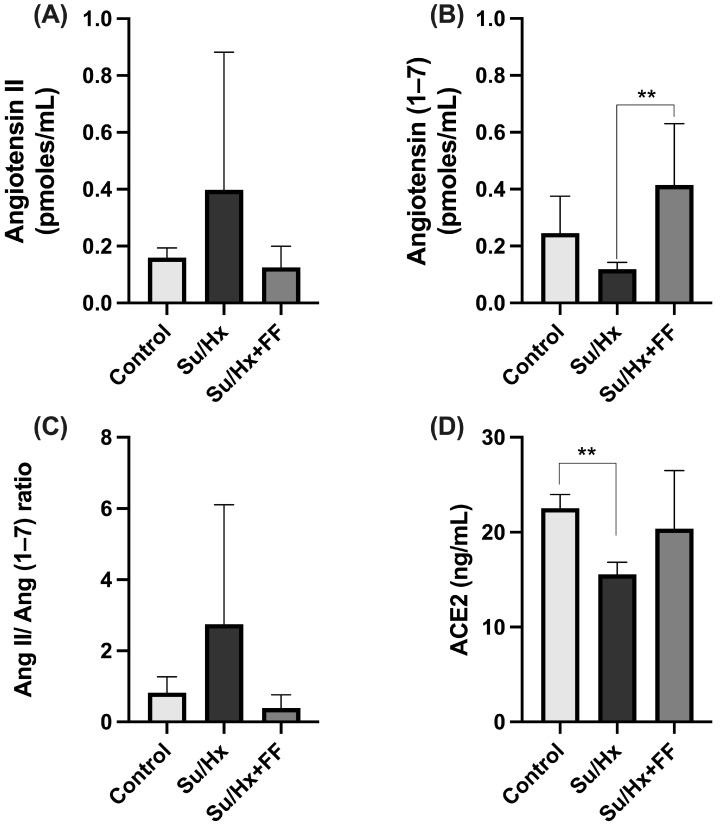
Fenofibrate modulates renin–angiotensin system components in the right ventricle in PAH. (**A**) Angiotensin II (Ang II) concentrations. (**B**) Angiotensin (1–7) (Ang (1–7)) concentrations. (**C**) Ang II/Ang (1–7) ratio. (**D**) Angiotensin-converting enzyme 2 (ACE2) concentrations. Data are presented as mean ± SD. Differences were tested by one-way ANOVA followed by Tukey’s multiple comparisons post hoc test; *p* ≤ 0.05 was considered statistically significant. ** *p* < 0.01 vs. Control.

No significant changes in Ang II concentration were observed in cardiac tissue, as shown in Figure 9A. On the other hand, Ang (1–7) levels were significantly higher in the Su/Hx + FF group compared to the Su/Hx group (*p* = 0.0043; Figure 9B). Regarding the Ang II/Ang (1–7) ratio, a non-significant trend toward normalization was observed in the Su/Hx + FF group (Figure 9C). Finally, ACE2 levels were significantly lower in the Su/Hx group compared to the control group (*p* = 0.0062). This decrease was partially attenuated by FF administration, although it did not reach statistical significance compared to the Su/Hx group (Figure 9D).

### 2.10. Effect of Fenofibrate Administration on Nitrate (NO_3_^−^) and Nitrite (NO_2_^−^) Concentrations in the Right Ventricle in a Su/Hx-Induced PAH Model

**Figure 10:**
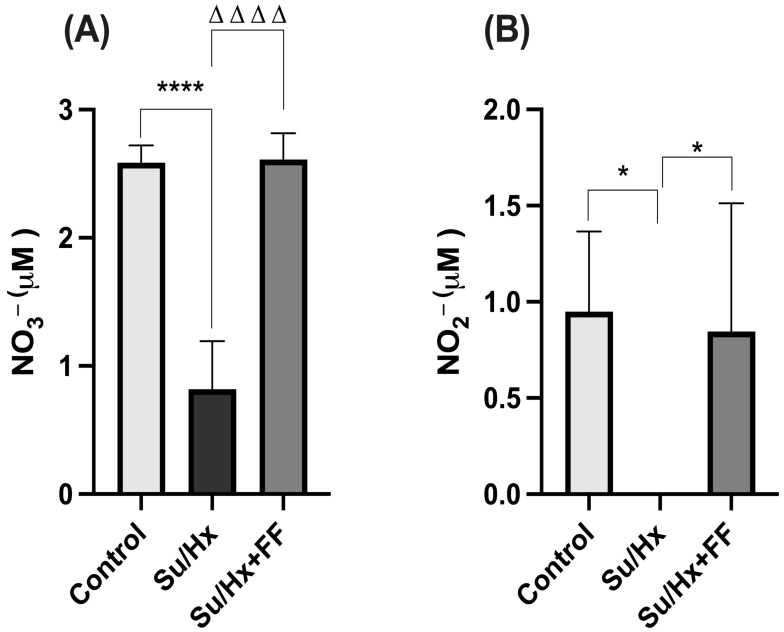
Fenofibrate preserves nitrate (NO_3_^−^) and nitrite (NO_2_^−^) concentrations in the right ventricle in PAH. (**A**) NO_3_^−^ concentrations in the right ventricle. (**B**) NO_2_^−^ concentrations in the right ventricle. Data are presented as mean ± SD. Differences were tested by one-way ANOVA followed by Tukey’s multiple comparisons post hoc test; *p* ≤ 0.05 was considered statistically significant. * *p* < 0.05, **** *p* < 0.0001 vs. Control; ΔΔΔΔ *p* < 0.0001 vs. Su/Hx.

In the right ventricle, a substantial decrease in NO_3_^−^ concentrations was observed in the Su/Hx group compared to the control group (*p* < 0.0001). Notably, FF administration preserved NO_3_^−^ concentrations compared to the Su/Hx group (*p* < 0.0001; Figure 10A).

NO_2_^−^ concentrations were undetectable in the Su/Hx group (Su/Hx vs. control, *p* = 0.0162; Figure 10B). In contrast, FF administration preserved NO_2_^−^ concentrations compared to the Su/Hx group (*p* = 0.0308; Figure 10B).

## 3. Discussion

In the present study, we evaluated the effect of FF on the development of PAH by modulating the RAS. Our findings indicate that prophylactic administration of FF in a Su/Hx-induced PAH rat model, which shares pathophysiological features with human PAH, confers a protective effect against disease progression. Furthermore, we observed that FF decreased Ang II concentrations and the Ang II/Ang (1–7) ratio in lung tissue, while preserving Ang (1–7) and ACE2 levels in the RV. Similarly, in the FF administration group, MAS receptor and ACE2 were highly expressed, whereas AT1R was more represented in the Su/Hx group in pulmonary arterioles. It is important to note that FF administration did not affect body weight gain or triglyceride concentrations (Supplementary Figures S1 and S3).

Notably, we confirmed that rats treated with Su/Hx developed PAH. Su/Hx induced RV hypertrophy, as evidenced by the increase in the Fulton index, consistent with previous findings reported by our group using the MCT model [6], as well as the hypertrophy findings observed in the Su/Hx model by Sitapara et al., who similarly reported hemodynamic parameters comparable to those observed in our study [41]. Also, we observed thickening of the pulmonary arterioles’ wall consistent with other Su/Hx models previously reported, which were used as sources for the development of our experimental model [41,42,43,44]. Although no animal model fully replicates all the pathophysiological features of human PAH [45] the Su/Hx model is currently considered the most robust and clinically relevant, as it reproduces several alterations observed in patients with PAH, such as plexiform lesions, pulmonary vascular remodeling, and molecular alterations [46,47,48]. This supports the use of Su/Hx as a robust model for studying pathogenetic mechanisms and evaluating therapeutic interventions.

Following our hypothesis, FF administration prevented the development of these pathological changes. FF prevented the development of RV hypertrophy compared to the untreated group (mean 0.3082 vs. 0.5112, respectively). Additionally, reductions in hemodynamic parameters, such as RVSP (mean 46.91 vs. 79.65 mmHg) and mPAP (mean 45.01 vs. 31.13 mmHg), were observed, along with an approximately 50% decrease in pulmonary arterioles wall thickening. Notably, the values of these parameters in the FF administration group were very similar to those of the control group. In this context, and to our knowledge, a study has previously explored the effects of FF in a monocrotaline-induced PAH model [7]. In the study by Golhotra et al., FF prevented RV hypertrophy, as assessed by the Fulton index, reduced RVSP, and prevented medial wall thickening [7]. Moreover, the dose of FF used in that study was higher than ours (120 mg/kg vs. 100 mg/kg); both results are consistent and similar. These findings support a protective role for FF in both MCT- and Su/Hx-induced PAH models in rats. Also, we found that FF administration decreased PAEa, which is partially associated with a 50% reduction in pulmonary wall thickening, improving its elasticity; consequently, lower pressure is required for blood to pass from the heart to the lungs through the proximal arteries and SPAP, the pulsatile component of the pulmonary circulation, is reduced. In contrast, in the distal pulmonary arteries, FF improves mPAP, the resistive component of the pulmonary circulation, and restores constant blood flow in the pulmonary vascular network [49,50]. On the other hand, in our Su/Hx-treated rats we observed a transition point between adaptive and non-adaptive RV failure, since these animals showed greater inotropy reflected in the increase in RVDP and maximum dp/dt, accompanied by the rise in the Fulton index, demonstrating hypertrophy of cardiac tissue in response to the pressure overload caused by PAH; together, these modifications in ventricular systolic function and RV morphology occur to maintain cardiac output without drastically altering RVDP, which is characteristic of the adaptation phase [51]. However, the minimum dp/dt increased, indicating a lower relaxation capacity compared to the control group, probably due to the increase in afterload [52]. In this regard, it has been reported that heart failure occurs under conditions of high afterload [53]. Furthermore, compensatory mechanisms to maintain RV contractility may not be sufficient to counteract afterload in these animals, since uncoupling of the RVPAC appears to be a consequence of poor RV adaptation to pressure overload [54]. It is worth mentioning that optimal RVPAC values are in the range of 1.5 to 2, and values lower than 0.8, such as those present in PAH, indicate an increase in afterload and a worse prognosis of the disease [55]. However, treatment with FF significantly improves the Fulton index, RVDP and minimum dp/dt. Although the increase in RVPAC is not significant in rats treated with FF, it should be noted that such treatment stops the progression to RV failure. On the other hand, no significant differences were observed in heart rate (Supplementary Material, Supplementary Figure S2), which is consistent with previous studies [56,57,58]. Although systemic blood pressure was not measured, clinical and preclinical studies focusing on RAS modulation have shown that such modulation can improve pulmonary hemodynamics without altering systemic blood pressure [56,57,58].

Furthermore, we investigated whether these effects were associated with RAS modulation. In our study, we observed increased Ang II levels and the Ang II/Ang-(1–7) ratio; however, these changes were attenuated in the FF group. Likewise, a reduction in Ang-(1–7) levels was observed in the lungs of the Su/Hx group compared to the control group. In the RV, decreased Ang-(1–7) levels were detected in the Su/Hx group, which were restored after FF administration. However, regarding ACE2 levels, FF administration failed to restore its expression, suggesting that Ang-(1–7) production is not exclusively dependent on ACE2 expression. Besides ACE2, other enzymes such as neprilysin, prolyl carboxypeptidase, and prolyl endopeptidase also participate in its synthesis [27,59]. In particular, it has been documented that neprilysin promotes Ang-(1–7) production, and that its pharmacological inhibition considerably reduces tissue levels of Ang-(1–7), confirming its relevant role in this pathway [59]. As such, neprilysin activity may compensate for the absence of significant ACE2 regulation and explain the restoration of Ang-(1–7) levels in the right ventricle of the Su/Hx + FF group. However, analysis of other components of the RAS system was outside the scope of this study. We also performed immunohistochemical localization of the AT1R and MAS receptors, as well as ACE2, in pulmonary arterioles. FF prevented AT1R overexpression and preserved the expression of MAS and ACE2. Our findings are consistent with previous studies that have shown that FF modulates several RAS components [19,22,26,29]. In a model of metabolic syndrome and myocardial ischemia, FF significantly reduced myocardial Ang II levels and AT1R expression, which was associated with improved endothelial function. Concurrently, FF preserved ACE2 expression and Ang-(1–7) levels, as well as the expression of AT2R and MAS receptors [34,37]. Moreover, in a murine model, FF prevented the development of Ang II-induced systemic arterial hypertension [40]. These findings support the role of FF as a positive modulator of the non-classical RAS axis, associated with anti-inflammatory, vasodilator, and antifibrotic effects, as well as its negative modulator role of the classical RAS axis, which is associated with opposing effects [5,27]. In the context of PAH, the results of this study are consistent with several studies that have focused on exploring the contribution of the RAS to the pathophysiology of PAH. In patients with PAH, a significant increase in plasma Ang II levels has been reported, accompanied by a decrease in Ang-(1–7) and Ang-(1–9) and a decrease in ACE2 activity [9,10]. Simultaneously, increased ACE activity and AT1R overexpression have been described in lung tissue, which could promote vasoconstriction and vascular remodeling [9]. Consistent with these clinical findings, PAH animal models have shown similar findings. For example, in MCT-induced PAH, it has been observed that increased Ang II and decreased Ang-(1–7) levels are present in both the lung and plasma [60]. Fried et al. demonstrated Ang II/AT1R axis activation in a nicotine-induced murine PAH model [24]. Additionally, Morrell et al. reported increased ACE activity in the RV following chronic hypoxia [23]. This imbalance has been associated with increased PASMC proliferation, vascular remodeling, RV hypertrophy, cardiac remodeling, and shorter survival [23,25]. On the other hand, it has been shown to promote the alternative RAS axis and reduce Ang II, ACE, and AT1R expression. It has exhibited clinically relevant effects, including attenuation of RVSP, RV hypertrophy, and both vascular and ventricular remodeling [20,22,28]. Furthermore, a decrease in PASMC proliferation has been reported, which may attenuate the development of RV dysfunction and improve RV pressure [1]. For example, in a monocrotaline-induced pulmonary hypertension model, overexpression of Ang–(1–7) exerted a cardioprotective role [21]. The main findings showed that either ACE2 overexpression, the enzyme responsible for generating Ang-(1–7), or Ang-(1–7) itself, protected the lungs from pulmonary hypertension [21]. Specifically, Ang-(1–7) overexpression attenuated the increase in RVSP, prevented RV hypertrophy, reduced medial wall thickness, and partially decreased fibrosis, mainly by improving pulmonary hemodynamics [21]. Although we did not perform a formal correlation analysis, our findings suggest a close relationship between structural remodeling and RAS imbalance. In the Su/Hx group, right ventricular hypertrophy and pulmonary arterial wall thickening were accompanied by increased Ang II levels, a higher Ang II/Ang-(1–7) ratio, and reduced ACE2 expression. In contrast, FF administration attenuated these structural alterations in parallel with the preservation of Ang-(1–7) levels and MAS receptor expression. These findings support the pathogenetic role of the classical RAS axis (ACE-Ang II-AT1R) in PAH progression and emphasize the therapeutic potential of strategies focused on reestablishing balance to the alternative axis (ACE2-Ang-(1–7)-MAS/AT2R), such as FF treatment.

Additionally, we explored the potential involvement of additional signaling pathways associated with vascular remodeling and endothelial function. Specifically, we evaluated the expression of *Bmpr2* and *Smad5*, critical components that have been implicated in PAH pathogenesis [61]. In our study, Su/Hx exposure significantly reduced lung tissue *Bmpr2* and *Smad5* expression, whereas FF administration partially restored their levels. These findings suggest that FF may exert pleiotropic effects in PAH by modulating not only the RAS but also the BMP signaling pathway, potentially contributing to the attenuation of pulmonary vascular remodeling. Previous studies have shown that reduction in *Bmpr2* signaling promotes proliferation and resistance to apoptosis in PASMC, and that restoration of *Bmpr2*/*Smad* signaling has a protective effect [11,12].Therefore, the partial recovery of *Bmpr2* and *Smad5* observed in our model may contribute to the vasoprotective effects associated with FF administration.

Finally, we investigated the protective effects of FF on nitric oxide production. NO_3_^−^ concentrations were significantly reduced in the RV of the Su/Hx group. Similarly, FF administration preserved NO_2_^−^ concentrations in lung tissue and the RV. These findings are consistent with previous reports on impaired nitric oxide (NO) signaling in experimental models of PAH [16,60,62]. Interestingly, FF administration preserved NO_3_^−^ levels in the RV, suggesting a potential role in maintaining NO bioavailability. This is consistent with studies showing that FF improves endothelial function and increases NO production, likely through the upregulation of eNOS and the inhibition of oxidative stress [16,37,63,64].

In this study, results suggest that the main effect of FF is on the pulmonary vasculature, and the changes observed at the cardiac level may be related to this vascular improvement. However, we cannot exclude that FF may exert direct effects on the myocardium, the characterization of which would require more specific experimental approaches, such as the pulmonary artery banding (PAB) model, which would allow a detailed assessment of direct cardiac effects [65]. On the other hand, although FF administration demonstrated a beneficial impact in the experimental model of PAH, its participation in different pathways and effects on other tissues, which were not assessed in this study, cannot be ruled out. Additionally, not all RAS components, for example, ACE and neprilysin, were quantified, which could limit their comprehensive characterization. Its therapeutic action is likely to involve additional yet uncharacterized mechanisms. Therefore, future research should adopt a broader approach that enables a deeper characterization of the molecular mechanisms underlying its protective effect.

## 4. Materials and Methods

### 4.1. Animals

Animal experiments in this study were performed with the approval of the National Institute of Cardiology Ethics Committee of Ignacio Chavez (Mexico) (INC/CICUAL/004/2023). All procedures complied with the Guide for the Care and Use of Laboratory Animals (2011) [66], Guidelines for the Euthanasia of Animals (2020) [67] and the Norma Oficial Mexicana NOM-062-ZOO-1999 [68].

**Figure 11:**
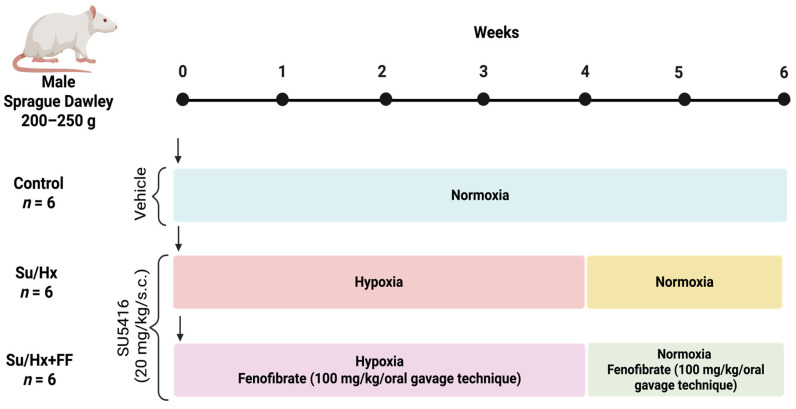
Experimental design.

Male Sprague Dawley rats (200–250 g) were used and housed under controlled conditions with a 12:12 light-dark cycle and an environmental temperature of 25 ± 2 °C. Animals were randomly allocated into three groups (*n* = 6 per group): (1) control (Control): maintained under normoxic conditions for six weeks; (*2*) SU5416/Hypoxia (Su/Hx): rats received a single subcutaneous dose of SU5416 (20 mg/kg), followed by exposure to hypoxia for 4 weeks, and then 2 weeks of normoxia; and (*3*) Su/Hx plus FF (Su/HX + FF): rats received the same treatment as the Su/Hx group, plus daily intragastric administration of FF (100 mg/kg/oral gavage technique) for six weeks. A schematic overview of the experimental design is in Figure 11. Rats were provided with commercial food and water ad libitum. Animals were euthanized under deep surgical anesthesia induced by intraperitoneal injection of pentobarbital sodium 70 mg/kg combined with heparin 1.7 U/kg. After induction and confirmation of the absence of reflexes, an open chest was performed. The heart and lungs were then collected while the animals remained in deep anesthesia, ensuring that they never regained consciousness and that euthanasia was achieved as part of the terminal procedure.

### 4.2. SU5416 and Fenofibrate Preparation

FF was administered at a dose of 100 mg/kg/day, prepared by dissolving in distilled water. The dose of FF used was selected based on previously published studies [7,34,37]. SU5416 (Cayman^®^ Chemical, Ann Arbor, MI, USA) was diluted in a solution composed of 100 μL of DMSO, 3 mL of propylene glycol (polyethylene glycol), and 100 μL of Tween 80; the final volume was adjusted to 10 mL. For each 5 mg of SU5416, 1 mL of this solution was used for dilution.

### 4.3. Induction of Pulmonary Arterial Hypertension

PAH was induced by the SU5416 plus hypoxia model (Su/Hx). A single subcutaneous dose of 20 mg/kg of SU5416 was administered. In addition to SU5416 administration, rats were exposed to continuous hypoxia for 4 weeks. Three rats were placed in a chamber (32 × 47 × 20 cm), which was introduced into a hypobaric chamber. This chamber was equipped with a vacuum-pressure gauge (Metron, Mexico City, Mexico to simulate an oxygen restriction equivalent to 76 mmHg (corresponding to an altitude of approximately 2500 m above sea level). The sealed compartment was connected to a Welch DuoSeal 1400B-01 two-stage belt-driven vacuum pump,1/3 hp motor, 580 rpm (Welch, Mt Prospect, IL, USA), capable of achieving a high vacuum (1 × 10^−4^ Torr) with a pumping speed ranging from 25 to 650 L/min (0.9 to 23 cfm). The animal model was developed based on previously described protocols [42,43,44,69].

### 4.4. Hemodynamic Parameters

After 6 weeks, animals were anesthetized by intraperitoneal injection of pentobarbital sodium (70 mg/kg) and heparin (1.7 U/kg). A tracheotomy was performed to introduce an endotracheal cannula connected to a small animal respirator (Model 683, Harvard Apparatus, Holliston, MA, USA), which was calibrated to a tidal volume of 1 cm^3^ with a respiratory rate of 65 breaths per minute. A lateral right thoracotomy was performed to expose the heart. A Mikro-Tip SPR-869 pressure-volume catheter transducer (Millar instruments, Houston, Tx, USA) was inserted into the right ventricular cavity. Digital hemodynamic recording was performed using the MPVS Ultra system and LabChart software, version 8. A 10 min stabilization period was allowed before recording hemodynamic parameters for 20 min. The hemodynamic variables measured were: mPAP, RVSP, RV end systolic pressure, RV end diastolic pressure, RVDevP, end systolic pressure volume relationship (ESPVR), arterial elastance (Ea), inotropy by dp/dt max and lusitropy by dp/dt min.

From the previously measured variables, the estimation of pulmonary hemodynamics was assessed, where SPAP, PAEa, and Ees were derived from RV end-systolic pressure, Ea, and ESPVR, respectively. Meanwhile, right ventricular-pulmonary arterial coupling (RVPAC) is the ratio between Ees/PAEa [49]. The mPAP was determined by the Chemla D method (2004) [50].

### 4.5. Evaluation of Right Ventricular Hypertrophy

Right ventricular (RV) hypertrophy was assessed using Fulton’s index [70]. The RV was separated from the left ventricle (LV) and the interventricular septum (IVS). The ratio of RV weight to LV plus IVS (RV/LV + IVS) (Fulton’s Index) was calculated using an analytical balance. The heart tissue was immediately frozen in liquid nitrogen and stored at −70 °C for further analysis.

### 4.6. Immunohistochemistry Analysis of Wall Thickness

The left lungs were used for histopathological analysis. The lungs were washed with saline solution to remove blood and then fixed by intratracheal perfusion with 4% neutral buffered formalin. The tissues were embedded in paraffin and sectioned at a thickness of 4 μm using a Leica Biosystems microtome (Deer Park, IL, USA). Immunohistochemical staining for α-smooth muscle actin (α-SMA) was performed following the manufacturer’s instructions in the Vectastain^®^ Universal Quick Kit, Peroxidase, R.T.U (Vector Laboratories, Newark, CA, USA). For each slide, 80 to 100 arterioles were selected specifically with an external diameter < 100 µm and analyzed to determine medial wall thickness using ZEN software (Carl Zeiss, Marly-le-Roi, France). Arterioles were micrographed at 40× magnification, and the percentage of wall thickness was calculated using the following formula: (2 × medial wall thickness/external diameter) × 100. Images were captured using a Biotek Lionheart FC automated live imaging microscope (Agilent Technologies, Santa Clara, CA, USA) CA, USA). The study was conducted blindly.

### 4.7. Concentrations of Angiotensin II and Angiotensin 1–7 in Lung and Right Ventricle

The concentrations of Ang II and Ang 1–7 in lung and RV (50 μL of sample) were simultaneously determined using the high-performance liquid chromatography (HPLC) (Beckman, System Gold, Urbana, IL, USA) [71]. Samples were deproteinized with cold methanol, centrifuged at 16,000× *g* for 15 min at 10 °C, and cold 0.1 M sodium hydroxide was added. Concentrations were expressed in pmol/mL and calculated using a standard curve.

### 4.8. Determination of Angiotensin-Converting Enzyme 2 by ELISA

ACE2 concentrations in lung tissue and RV heart were quantified using an ELISA assay, according to the manufacturer’s protocol (Rat ACE2 ELISA Kit, MyBioSource^®^ San Diego, CA, USA; Cat. No. MBS2516179).

### 4.9. Immunohistochemistry

Lung tissue sections, 4 µm thick, were placed on silanized slides to perform immunohistochemical analysis. After deparaffinization and rehydration, antigen retrieval was performed using a citrate buffer solution (ImmunoDNA Retriever Citrate, Bio SB, Santa Barbara, CA, USA) via heat-induced epitope retrieval (HIER). Endogenous peroxidase activity was quenched by incubation with 0.3% hydrogen peroxide. Non-specific binding was blocked using Background Sniper (Biocare Medical, Pacheco, CA, USA). The lung sections were then incubated overnight at room temperature with the following primary antibodies diluted 1:200: MAS1 (G-1) mouse monoclonal IgG1k antibody (sc-390453, Santa Cruz Biotechnology, Dallas, TX, USA), and ACE2 (E-11) mouse monoclonal IgG1k antibody (sc-390851, Santa Cruz Biotechnology, Dallas, TX, USA). Antibody diluted 1:50 angiotensin receptor/AT1/AGTR1 (G-3) mouse monoclonal IgG 1 k antibody (sc-515884, Santa Cruz Biotechnology, Dallas, TX, USA). After primary antibody incubation, sections were treated with the mouse/rabbit polydetector DAB HRP detection system (Bio SB). Immunoreactivity was visualized using the diaminobenzidine (DAB) substrate (Merck KGaA, Darmstadt, Germany), and the sections were counterstained with hematoxylin. At least ten independent fields from four different sections were analyzed using a Biotek Lionheart FC automated live imaging microscope (Agilent Technologies, Santa Clara, CA, USA) at 40× magnification. The positive area was quantified using the Color Deconvolution version 1.7 plugin of Fiji software (NIH, Badesta, MD, USA) on ten sections per animal. The results were presented as cell area positive for ECA, MAS and AT1.

### 4.10. Determination of Nitrate (NO_3_^−^) and Nitrite (NO_2_^−^) Levels by Colorimetric Assay Kit

NO_3_^−^ and NO_2_^−^ concentrations were determined in lung tissue and RV using a colorimetric assay, following the manufacturer’s instructions (Nitrite/Nitrate Colorimetric Assay Kit, Cayman Chemical, Ann Arbor, MI, USA; Cat. No. 780001).

### 4.11. Bmpr2 and Smad5 mRNA Expression by RT-qPCR

Total RNA was extracted from 50 mg of lung tissue using QIAzol Lysis Reagent (Qiagen, Hilden, Germany), according to the manufacturer’s instructions. RNA concentration and purity were determined. Subsequently, 1000 ng of total RNA was reverse transcribed into cDNA using the QuantiTect Reverse Transcription Kit (Qiagen, Hilden, Germany). Quantitative PCR was performed using the QuantiNova SYBR^®^ Green PCR Kit (Qiagen, Hilden, Germany) on a LightCycler^®^ 480 II System (Roche, Basel, Switzerland). Expression levels were calculated using the 2^−ΔΔCt^ method. GAPDH was used as a reference gene for normalization. The primer sequences used are listed in Table 1. 

## 5. Conclusions

In conclusion, these findings indicate that FF exerts cardiopulmonary protective effects in Su/Hx-induced PAH, including the prevention of RV hypertrophy, the reduction in mPAP and RVSP, and the attenuation of pulmonary arterial wall thickening. Our results suggest that these effects may be mediated by the modulation of the RAS, along with other protective mechanisms, such as a beneficial influence on nitric oxide metabolism. However, further studies are required to clarify the mechanisms underlying these protective effects, which could provide valuable insights into the therapeutic potential of FF in PAH. Moreover, this study suggests that drug repositioning, such as the use of FF, represents a promising strategy for managing PAH, as well as identifying new therapeutic targets.

## Figures and Tables

**Table 1:** Primer sequences used for RT-qPCR analysis.

Gene	Direction	Sequences
*Bmpr2*	Forward	gagccctccctggacttg
	Reverse	atatcgaccccgtccaatc
*Smad5*	Forward	gcctatggacacaagcaaca
	Reverse	aggcaacaggctgaacatct
GAPDH	Qiagen (Cat. No. PPM02946E)	

## Data Availability

The original contributions presented in this study are included in the article/Supplementary Materials. Further inquiries can be directed to the corresponding authors.

## Supplementary Materials

The following supporting information can be downloaded at: https://www.mdpi.com/article/10.3390/ijms262110251/s1.
