# Supplementary material for: Fenofibrate as a Modulator of the Renin–Angiotensin System in Su/Hx-Induced Pulmonary Arterial Hypertension
Source: Int J Mol Sci. 2025 Oct 22;26(21):10251. doi: 10.3390/ijms262110251 (PMC12610544; doi:10.3390/ijms262110251)
Supplement: Supplementary file 1 [file ijms-26-10251-s001.zip › ijms-3894173-supplementary.pdf]

We evaluated whether FF administration induced changes in body weight.

The final body weight of the Control, Su/Hx, and Su/Hx+FF groups showed no statistically significant differences (Supplementary Figure 1A). Similarly, no significant differences were found in total body weight gain (Supplementary Figure 1B).

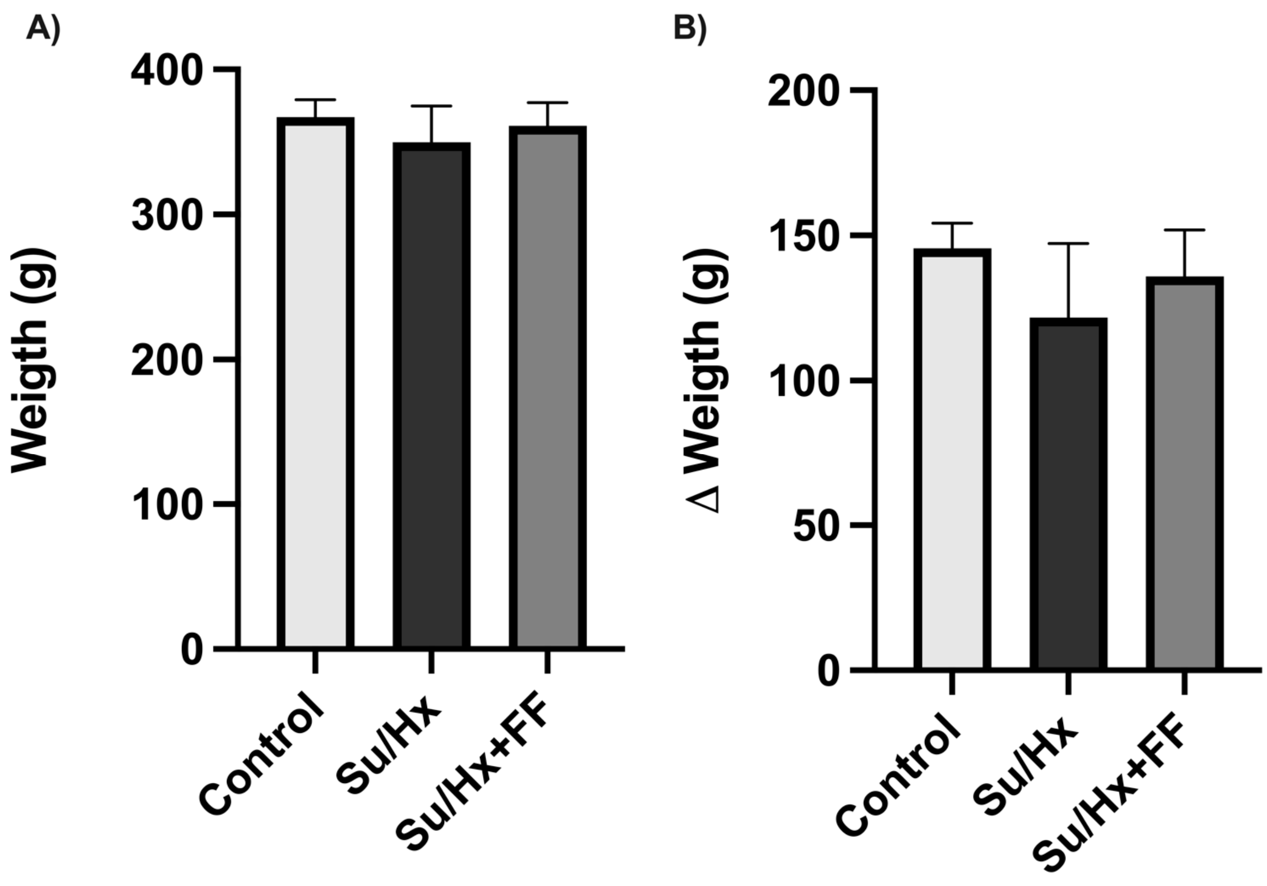

Supplementary Figure S1. (A) Final body weight and **(B)** Total body weight gain. Data are presented as mean ± SD. Differences were tested by one-way ANOVA followed by Tukey's multiple comparisons post hoc test; $p \leq 0.05$ was considered statistically significant.

We evaluated whether FF administration induced changes in the heart rate.
The heart rate of the Control, Su/Hx, and Su/Hx+FF groups showed no statistically significant differences (Supplementary Figure 2).

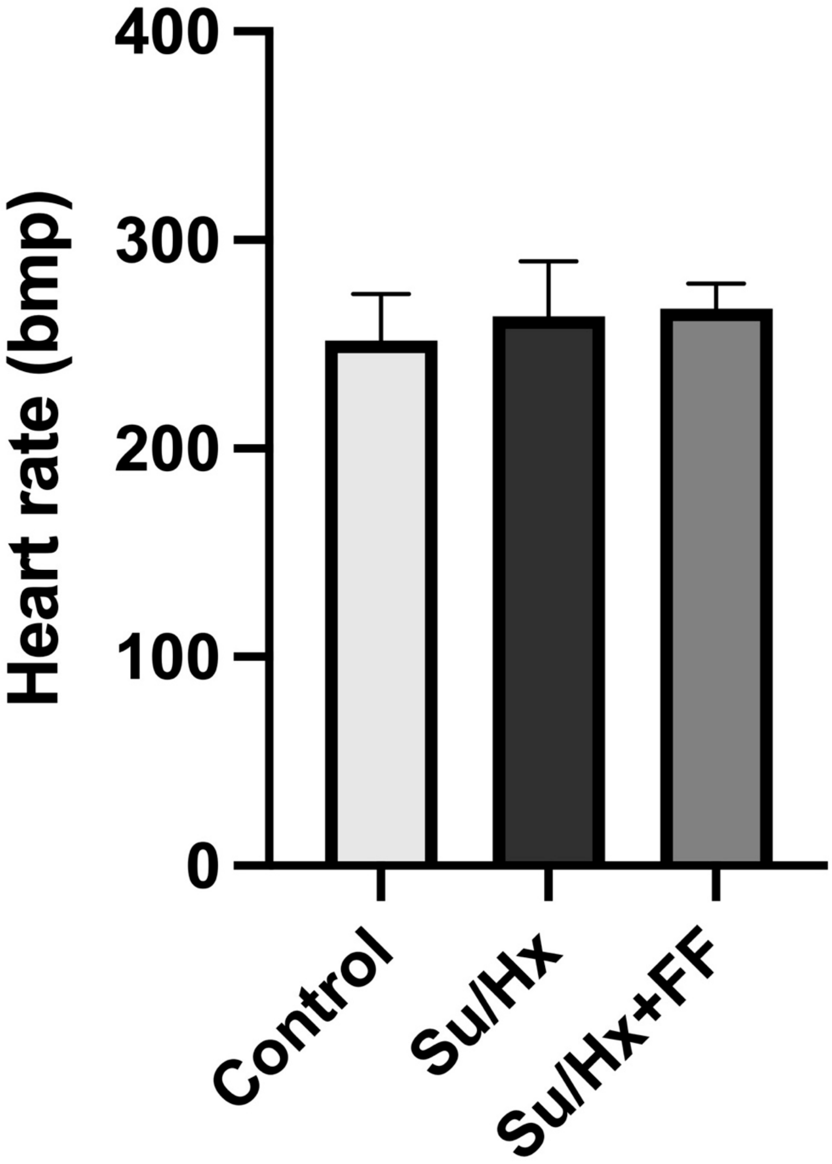

Supplementary Figure S2. Heart rate. Data are presented as mean ± SD. Differences were tested by one-way ANOVA followed by Tukey's multiple comparisons post hoc test; $p \leq 0.05$ was considered statistically significant.

We also evaluated whether FF administration induced changes in triglyceride concentrations.

No statistically significant differences in triglyceride concentrations were observed among groups (Supplementary Figure 3).

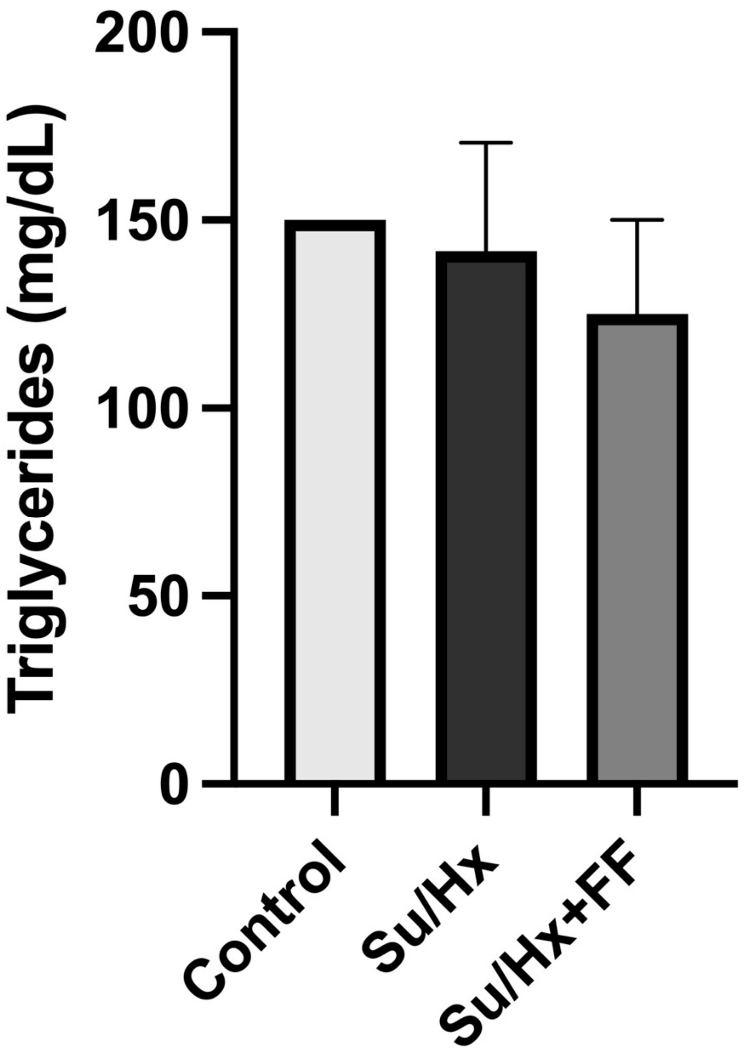

Supplementary Figure S3. Triglyceride concentrations. Data are presented as mean ± SD. Differences were tested by one-way ANOVA followed by Tukey's multiple comparisons post hoc test; $p \leq 0.05$ was considered statistically significant. n= 3. Triglyceride concentrations will be determined using the colorimetric enzymatic method Triglycerides-LQ (Spinreact®, Spain), following the manufacturer's specifications.
